# Supplementary material for: Complexome profiling on the Chlamydomonas lpa2 mutant reveals insights into PSII biogenesis and new PSII associated proteins
Source: J Exp Bot. 2021 Aug 26;73(1):245–62. doi: 10.1093/jxb/erab390 (PMC8730698; doi:10.1093/jxb/erab390)
Supplement: erab390_suppl_Supplementary_Dataset_S1 [file erab390_suppl_supplementary_dataset_s1.zip › Supplemental Dataset 1 - Excel List and all profiles/plots/_Cre10.g465650.html]

Trivial name:   
  
Euclidean distance: 4110.59  
Mean Intensity (WT): 0.00  
Mean Intensity (Mut): 114.18  
Distance: 36.00  
  
MapMan: development.unspecified  
  
p value of intensity sums Welch test: 0.4226
